# Effects of Mind–Body Exercises on Schizophrenia: A Systematic Review With Meta-Analysis

**DOI:** 10.3389/fpsyt.2020.00819

**Published:** 2020-08-13

**Authors:** Gao-Xia Wei, Lin Yang, Kellie Imm, Paul D. Loprinzi, Lee Smith, Xiangyang Zhang, Qian Yu

**Affiliations:** ^1^ Key Laboratory of Mental Health, Institute of Psychology, Chinese Academy of Sciences, Beijing, China; ^2^ Key Laboratory of Behavioral Science, Institute of Psychology, Chinese Academy of Sciences, Beijing, China; ^3^ Department of Psychology, University of Chinese Academy of Sciences, Beijing, China; ^4^ Department of Cancer Epidemiology and Prevention Research, Alberta Health Services, Calgary, AB, Canada; ^5^ Departments of Oncology and Community Health Sciences, Cumming School of Medicine, University of Calgary, Calgary, AB, Canada; ^6^ Keck School of Medicine, University of Southern California, Los Angeles, CA, United States; ^7^ Department of Health, Exercise Science and Recreation Management School of Applied Sciences, The University of Mississippi, Oxford, MS, United States; ^8^ Cambridge Centre for Sport and Exercise Sciences, Anglia Ruskin University, Cambridge, United Kingdom; ^9^ Center for Lifestyle and Mental Health, School of Psychology, Shenzhen University, Shenzhen, China; ^10^ Exercise and Mental Health Laboratory, School of Psychology, Shenzhen University, Shenzhen, China

**Keywords:** schizophrenia, neurological disorder, mind–body exercise, intervention, yoga

## Abstract

**Background:**

Mind–body exercises (MBEs) have been widely accepted as a complementary therapy for the patients with low exercise tolerance. Currently, the number of experimental studies investigating the effect of MBEs for improving symptoms in people with schizophrenia is increasing. However, results are inconsistent.

**Methods:**

We systematically reviewed and meta-analyzed the effects of mind–body exercises on schizophrenia. Seven electronic databases (Pubmed, Web of Science, PsycINFO, Embase, Cochrane Central Register of Controlled Trials [CENTRAL], CNKI and Wangfang) were screened through October 2019 and risks of bias of included studies were assessed in Review Manager 5.3.

**Results:**

Meta-analysis on 13 studies with 1,159 patients showed moderately significant effects in favor of mind–body exercise intervention to improve positive symptoms (SMD = 0.31; 95% CI 0.01 to 0.60; *p* = 0.04), negative symptoms (SMD = 0.37; 95% CI 0.14 to 0.60; *p* = 0.002), and depression (SMD = 0.88; 95% CI 0.63 to 1.13; *p*<0.00001). Meta-regression analysis revealed that the improvement in positive symptoms was positively associated with the frequency of intervention (*p* = 0.04), while a marginally significant correlation was observed between the improved negative symptoms and duration of each session (*p* = 0.06).

**Conclusions:**

This meta-analysis supports the therapeutic effects of MBEs to aid in the treatment of schizophrenia. Further studies need to incorporate rigorous design and large sample size to identify the optimal type and dose of mind–body exercise to inform clinical practices on MBEs’ recommendations for the management of schizophrenia symptoms.

## Introduction

Schizophrenia, as one of the chronic and severe mental illness, usually emerges between 16 and 30 years old, with its prevalence ranging from 0.33 to 0.75% globally (1). There are three types of symptoms that may be present in people with schizophrenia, including positive symptoms (delusions and hallucinations), negative symptoms (insufficient motivation, spontaneous reduction of speech, and social withdrawal), and cognitive symptoms (executive dysfunction, inattention, and working memory impairment) (2). These complex symptoms may also lead to a decline in social function and quality of life, a high degree of disability, and concurrent emotional diseases. It has become a public health issue because of its profound impact on family and society (3).

Since its pathology still remains elusive, treatments primarily focus on alleviating symptoms of the disease and include antipsychotic medications, psychosocial counseling, and coordinated specialty care (4). Although antipsychotic drugs can effectively improve certain positive symptoms, their beneficial effects on negative symptoms are limited (3, 5–8), and around 30% of patients are refractory to treatment (9). Moreover, antipsychotic drugs are connected with side effects including hesitation, retention, and transient leukopenia (10, 11). Prolonged use of antipsychotics may exacerbate the progression of cognitive impairment caused by schizophrenia (10, 11) and even lead to more adverse effects, which has been associated with impairments of the endocrine system (weight gain, hyperprolactinemia, and diabetes mellitus), the cardiovascular system (orthostatic hypotension), and the central nervous system (dystonia, akathisia, pseudoparkinsonism, and dyskinesia) (11). Psychosocial counseling and coordinated specialty care are typically adopted as a second line of treatment when antipsychotic medications fail to alleviate symptoms. These methods are expert-based and require a significant amount of time and cost heavily. Thus, researchers have attempted to seek low risk alternative therapies for people with schizophrenia.

Tai Chi, Yoga, and Qigong (including Baduanjin and Wuqinxi) are the three most popular mild to moderate intensity mind–body exercises (MBEs), and they have been increasingly accepted for treating patients with low exercise tolerance (12–18). MBEs are characterized by slow physical movement (stretching and relaxation of skeletal muscles) coordinated with abdominal breathing and meditative stage of mind (19, 20). These unique features have intrigued researchers and clinicians to extensively investigate the therapeutic effects of MBEs on diseases, particularly for those who are diagnosed with mental illnesses like schizophrenia (21). Indeed, the number of studies reporting beneficial effects of MBEs in schizophrenia is growing. However, findings are inconsistent: some studies showed that MBEs could be a useful add-on treatment for schizophrenia (22, 23), while others did not believe MBEs could offer more advantages over regular exercise or treatment as usual (24). Thus, a systematic review is needed to synthesize the existing literature. While there were five reviews on this topic, they focused on either one type of MBE (25), just negative symptoms (26, 27), qualitative synthesis (26) or MBEs-active control comparison (28), or included non-MBE studies (26), which make it difficult to provide an overview of MBE-induced effect on multiple symptoms of schizophrenia. Therefore, a comprehensive review with quantitative synthesis is necessary to systematically investigate the association between MBEs and a wide range of health outcomes in schizophrenia. Findings of this meta-analysis can identify knowledge gaps and provide researchers and clinicians with evidence-based recommendations so as to develop effective MBE treatments for schizophrenia patients.

## Methods

This study followed PRISMA guidelines (29) and Cochrane Collaboration’s recommendation (30) for systematic reviews and meta-analyses.

### Search Strategies

Five English databases (PubMed, Web of Science, PsycINFO, Embase, Cochrane Central Register of Controlled Trials (CENTRAL)) and two Chinese databases (CNKI, Wangfang) were systematically searched from their inception to October 1^st^ 2019. Literature search was detailed below:(((schizophrenia[Title/Abstract]) OR schizophrenic [Title/Abstract])) AND ((((((((((((mind–body[Title/Abstract]) OR mind body [Title/Abstract]) OR meditation[Title/Abstract]) OR meditative[Title/Abstract]) OR Tai Chi[Title/Abstract]) OR Taiji[Title/Abstract]) OR Qigong[Title/Abstract]) OR Baduanjin[Title/Abstract]) OR Wuqinxi[Title/Abstract]) OR Yoga[Title/Abstract]) OR Yogic[Title/Abstract]) OR Pilates[Title/Abstract]). Reference lists of identified studies were also screened.

### Eligibility Criteria

Firstly, studies (including randomized controlled trials and controlled trials with non-randomization) published in English and Chinese were considered eligible only if full-text articles could be retrieved. Secondly, subjects had to be aged 16 and above who were diagnosed with schizophrenia. Thirdly, to be eligible, the experimental group must involve at least one type of MBE (*e.g.*, Tai Chi, Qigong, or Yoga) alone or a combined training mode, whereas participants in the control group maintained their unaltered lifestyle or engaged in an active control condition like psychotherapy. Initially identified records were screened by two independent reviewers to remove duplicates and obviously irrelevant records. Then, potentially eligible full-text articles were read to determine if they met the eligibility criteria or not. Disagreements were discussed with a third reviewer author.

### Data Extraction and Management

Two reviewers used *a priori* developed data extraction forms to record all the information and extract data on patients independently in demographic data, methods, interventions, protocol as well as the outcomes.

### Risk of Bias in Individual Studies

Risk of bias of eligible studies was assessed in Review Manager 5.3 software and the criteria of Cochrane Handbook for Systematic Reviews and meta-analysis were followed (30). The quality of evidences were also assessed *via* GRADE (Grading of Recommendations Assessment, Development and Evaluation) system (31).

### Data Analysis

#### Assessment of Effect Size

Review Manager software was used for meta-analyses in random-effects model (30). Meta-analysis was only conducted if there were two or more pairs (experimental group *vs* control group) of comparisons on at least one health outcome (30). Standardized mean differences (SMD) with 95% confidence intervals (CI) were calculated as the difference in means between groups divided by the pooled standard deviation. SMD that reflects the magnitude of the overall effect size was categorized into: 1) Small = 0.2 to 0.5; 2) moderate = 0.5 to 0.8 and 3) large = 0.8 and above (32).

Levels of evidence were classified into five levels (strong evidence, moderate evidence, limited evidence, conflicting evidence, and no evidence) based on consistent findings, number of RCTs and risk of bias (33).

#### Assessment of Heterogeneity

I² statistic was used to identify between-study heterogeneity (low heterogeneity = 0–25%, moderate heterogeneity = 26–50%, substantial heterogeneity = 51–75%, and considerable heterogeneity = 76–100%) (30, 34).

#### Meta-Regression

Weighted meta-regressions were conducted for continuous, moderator variables like total duration of intervention, frequency of intervention, and duration of each session (35).

## Results

### Literature Search

**Figure 1:**
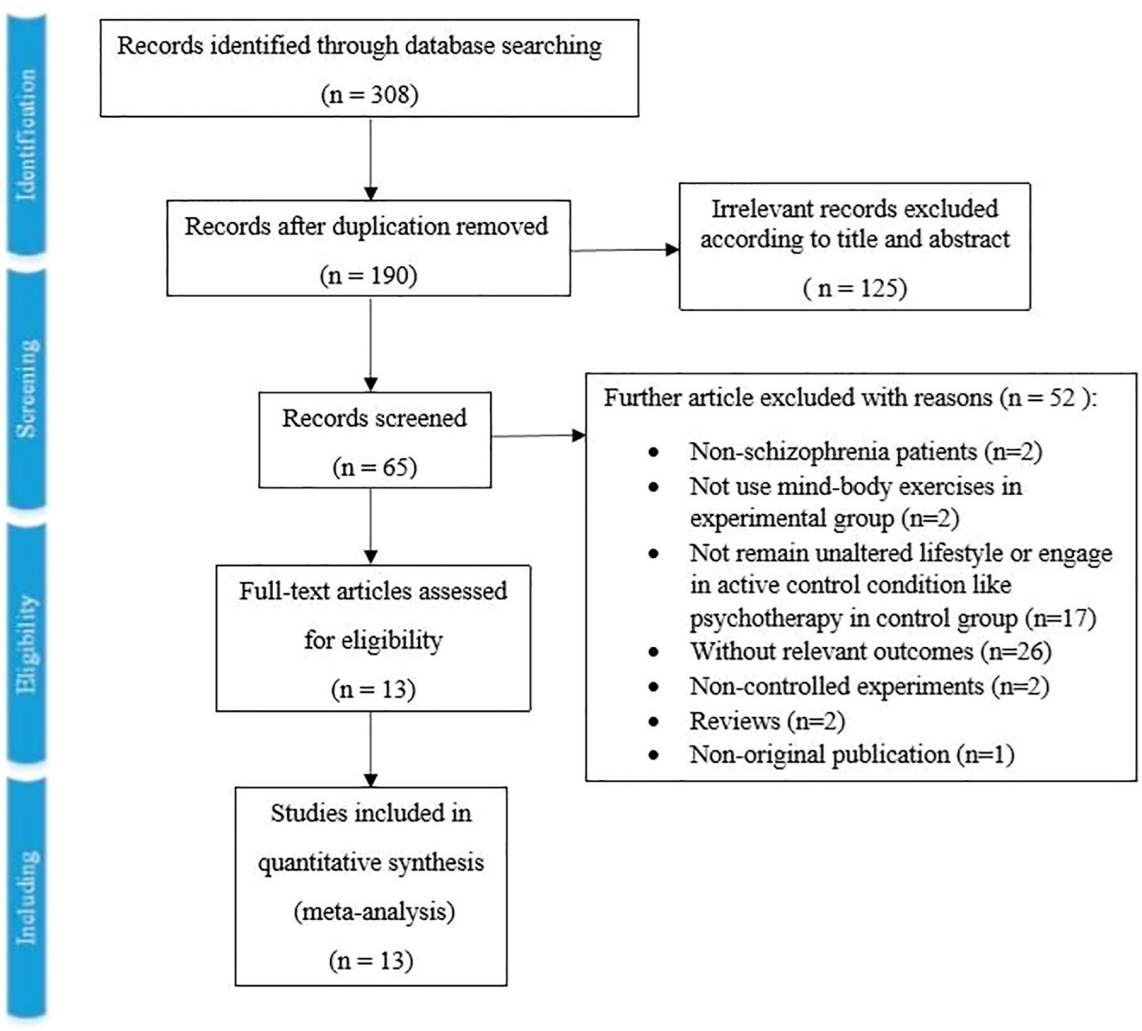
The Detailed Process of Study Selection.

As **Figure 1** showed, the literature search retrieved 308 records in total, and 118 records were excluded for duplication. Then, 190 records were screened by title and abstract, and 125 records were excluded for relevancy. Sixty-five full-text articles were screened, and 52 records were excluded with reasons (two included non-schizophrenia patients, two did not use MBEs in the experimental group, 17 did not remain unaltered lifestyle or engage in active control condition like psychotherapy in the control group, 26 without relevant outcomes, two non-controlled experiments, two reviews and one non-original publication). Finally, 13 studies with 1,159 patients were included in this systematic review and meta-analysis (22–24, 36–45).

### Study Characteristics

This systematic review and meta-analysis included 13 studies (11 randomized controlled studies (22–24, 36, 38–42, 44, 45), one non-randomized controlled study (37) and one quasi-experimental study (43). Characteristics of the participants, interventions, and outcome assessments are shown in **Table 1**.

**Table 1:** Descriptive Information of Included Studies.

Study	Age (mean or range)	Gender	Diagnostic; Disgnostic criteria	Setting	Intervention of control group (N)	Intervention of experimental group (N)	Cointervention	Duration of intervention	Study duration	Measurements of outcome
**Behere et al. (** 23 **)**	18–60	M:F = 32:12	Schizophrenia; DSM IV	Hospital outpatients	Treatment as usual (N = 17)	Yoga (N = 27)	Antipsychotic medication	Not mentioned	-1 month by yoga instructor - 2 month home practice	- PANSS - SOFS
**Bhatia et al.** **(** 44 **)**	>18	/	Schizophrenia; DSM IV	Hospital outpatients	Treatment as usual (N = 90)	Yoga (N = 104)	Not memtioned	60 min/day, 21 days by yoga instructor. Thereafter at home for 6 months.	-21 days by yoga instructor -6 months home practice	-Penn CNB -SANS -SAPS -GAF
**Duraiswamy et al. (** 22 **)**	18–55	M:F = 42:19	Schizophrenia; DSM IV	Hospital outpatients	Treatment as usual (N = 30)	Yoga (N = 31)	Antipsychotic medication	60 min/day, 5×/week for 3 weeks by yoga instructor. Thereafter at home for 3 months.	4 months	-PANSS -SOFS (AIMS) -WHOQOL-BREF - Shedding Rate: 33%
**Ho et al. (** 24 **)**	18–65	/	Schizophrenia; DSM IV-TR	Residing in a mental health rehabilitation hostel	Treatment as usual (N = 51)	Tai Chi (N = 51)	Not memtioned	45 min/day, 2×/week for 12 weeks by mental health professionals	-12 weeks by instructor -3 months (no practice)	-Chinese Version of the PANSS -Forward and backward digit spans test of the Chinese Wechsler Adult Intelligence Scale - Shedding Rate: 2%
**Manjunath et al. (** 40 **)**	31.1–31.7	M:F = 39:49	Schizophrenia; DSM IV	Hospital patients	Treatment as usual (N = 44)	Yoga (N = 44)	Antipsychotic medication	60 min/day, 5×/week	-2 weeks by yoga instructor -4 weeks yoga at home	-PANSS -HDRS -SAS - Shedding Rate: 32%
**Varambally et al. (** 39 **)**	30.6–32.8	M:F = 56:28	Schizophrenia; DSM IV	Hospital outpatients	Treatment as usual (N = 37)	Yoga (N = 47)	None	45 min/day, 25 days by yoga instructor. Thereafter 3 months of yoga at home.	4 months	-PANSS -SOFS - Shedding Rate: 21%
**Bhatia et al. (** 37 **)**	>18	/	Schizophrenia; DIGS	Hospital outpatients	Treatment as usual (N = 23)	Yoga (N = 23)	Antipsychotic medication	1 h/day, 21 days by yoga instructor.	21 days	Antomated computerized battery
**Rainbow et al. (** 38 **)**	41.02–62.72	M:F = 12:18	Schizophrenia; DSM IV-TR	Rehabilitation residency	Treatment as usual (N = 15)	Tai Chi (N = 15)	30-min daily morning stretching routine	1 h/session, twice a week by instructor.	6 weeks	-CMDT -SANSs -WHODAS-II - Shedding Rate: 20%
**Saeko et al. (** 41 **)**	>18	/	Schizophrenia; ICD-10	Hospital outpatients	Treatment as usual (N = 25)	Yoga (N = 25)	Antipsychotic medication	1 h/session, once a week by yoga instructor.	8 weeks	-PANSS -DIEPSS -FACT-Sz -EQ-5D
**Saeko et al. (** 45 **)**	39.2–70.8	M:F = 36:20	Schizophrenia; ICD-10	Hospital outpatients	Treatment as usual (N = 28)	Yoga (N = 28)	Antipsychotic medication	20 min/session, 24 sessions in all by yoga instructor.	12 weeks	-EQ-5D -GAF -MFES -PANSS -DIEPSS -TIP-Sz
**Kang et al. (** 42 **)**	18–60	M:F = 116:128	Schizophrenia; ICD-10	Community Health Center	Treatment as usual (N = 126)	Tai Chi (N = 118)	Antipsychotic medication	120 min/session, 2 sessions/month by instructor.	12 months	-PANSS -WHOQOL
**Funda and Mine (** 43 **)**	18–55	M:F = 73:27	Schizophrenia; Not mentioned	Mental health center	Treatment as usual (N = 50)	Yoga (N = 50)	Not mentioned	40 min/session, 5 sessions/week by yoga instructor.	6 weeks	-FROGS
**Elizabeth and Stephen (** 36 **)**	28.5–55.5	/	Schizophrenia; Not mentioned	Mental health center	Treatment as usual (N = 8)	Yoga (N = 10)	Not mentioned	45 min/session, 2 sessions/week by yoga instructor.	8 weeks	-PANSS -WHOQOF-BREF

DSM, The Diagnostic and Statistical Manual of Mental Disorders; GAF, Global Assessment of Functioning scale; PANSS, Positive and Negative Syndrome Scale; Penn CNB, University of Pennsylvania Computerized Neurocognitive Battery; SANS, Scale for Assessment of Negative Symptoms; SAPS, Scale for Assessment of Positive Symptoms; SOFS, Social and Occupational Functioning Scale; AIMS, Abnormal Involuntary Movement Scale; WHOQOL-BREF, World Health Organization Quality of Life-BREF; HDRS, Hamilton depression rating scale; SAS, Simpson angus scale for extrapyramidal side effects; DIGS, Diagnostic Interview for Genetic Studies; CMDT, Minnesota Rate of Manipulation Test; SANSs, Scale for the Assessment of Negative Symptoms; WHODAS-II, World Health Organization Disability Assessment Schedule; ICD-10, The International Classification of Diseases, 10th edition; DIEPSS, The Drug Induced Extrapyramidal Symptoms Scale; FACT-Sz, The Functional Assessment for Comprehensive Treatment of Schizophrenia; EQ-5D, The EuroQol-5 Dimensions; GAF, Global Assessment of Functioning; MFES, Modified Falls Efficacy Scale in Japanese; TIP-Sz, Targeted Inventory on Problems in Schizophrenia; FROGS, Functional Remission of General Schizophrenia Scale.

### Setting and Participant Characteristics

Among the 13 studies that were included, six originated from India (559 participants), three from China (376 participants), two from Japan (106 participants), one from Turkey (100 participants) and one from America (18 participants). Patients were recruited from hospitals, rehabilitation residencies, a community health center, and mental health centers.

Patients in five studies were diagnosed with schizophrenia according to DSM-IV, two with DSM-IV-TR, one with Diagnostic Interview for Genetic Studies (DIGS), and two with ICD-10. Two studies did not report the diagnostic criteria. Patients in one study were diagnosed with psychiatric disorders according to ICD-10. In the study of Bhatia et al., physical and mental comorbidities were: bipolar I disorder (n = 40), major depression disorder (n = 37) and cardiology (n = 68). Patients’ mean age ranged from 18 to 65 years. All trials included both males and female.

### Risk of Bias Within Studies

**Figure 2:**
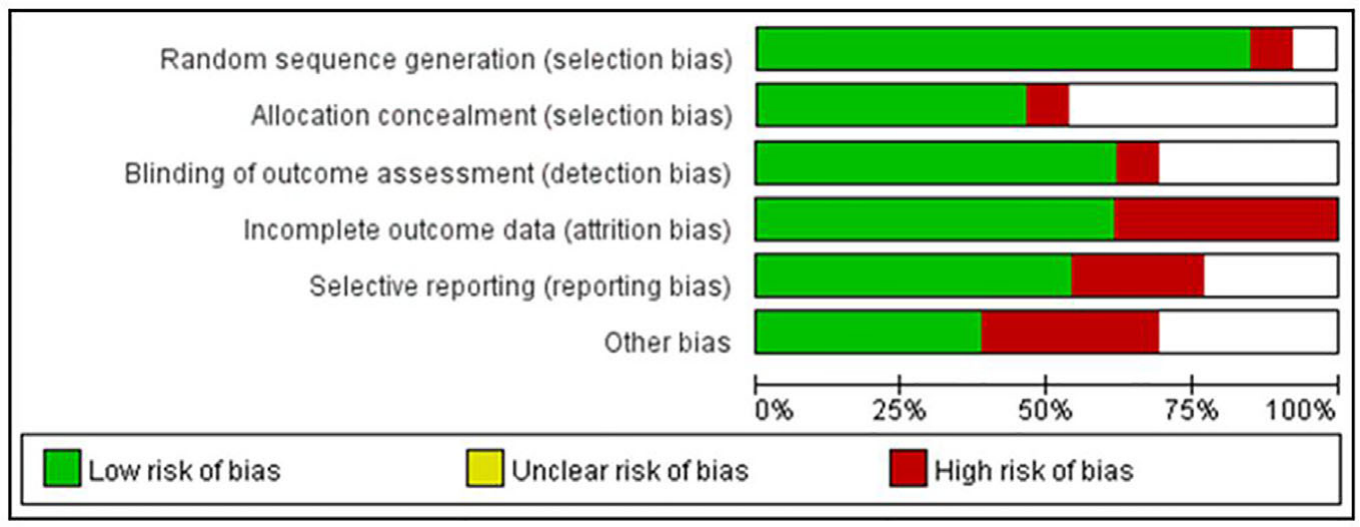
Risks of Bias within Studies.

**Figure 2** summarizes the risk of bias in the selected studies. It shows that the selected studies demonstrated low risk of bias except blinding of participants and personnel for its unfeasibility for conducting MBEs’ interventional study. Specifically, 11 studies were RCTs and used adequate random sequence generation. Only one study stated that assessors were not blinded, and one study had attrition bias (incomplete outcome data). Six studies had allocation concealment procedures. Three studies had been affected by reporting bias, and four had other potential sources of bias. Additionally, according to the assessment through GRADE system, five of 10 outcomes (positive and negative symptoms, depression, general psychopathology, and social function) were of moderate quality, while the others were of low quality (**Table 2**).

**Table 2:** Summary of findings *via* GRADE System.

**Mind–Body Exercises Compared with Treatment as Usual for Schizophrenia**						
**Patient or population: patients with schizophrenia. Settings: home, community, or hospital. Intervention: mind–body exercises. Comparison: treatment as usual.**						
**Outcomes**	Illustrative comparative risks* (95% CI)		Relative effect(95% CI)	No of Participants(studies)	Quality of the evidence(GRADE)	Comments
	Assumed risk	Corresponding risk				
	Treatment as Usual	Mind–body Exercises				
**Positive Symptoms**	The mean score ranged across control groups from −0.60 to 4.50.	The mean score in the intervention groups was 2.56 (0.12 to 6.00).	/	747	⊕⊕⊕⊝ **moderate**	
**Negative Symptoms**	The mean score ranged across control groups from −0.70 to 4.30.	The mean score in the intervention groups was 2.68 (0.70 to 7.71).	/	747	⊕⊕⊕⊝ **moderate**	
**General Psychopathology**	The mean score ranged across control groups from −1.75 to 2.70.	The mean score in the intervention groups was 3.71 (−0.60 to 13.30).	/	456	⊕⊕⊕⊝ **moderate**	
**Quality of Life** **(Physical Score)**	The mean score ranged across control groups from −6.25 to 2.50.	The mean score in the intervention groups was 7.71 (−0.42 to 12.25).	/	323	⊕⊕⊝⊝ **low**	
**Quality of Life** **(Psychological Score)**	The mean score ranged across control groups from −5.63 to 4.38.	The mean score in the intervention groups was 13.22 (2.08 to 22.5).	/	323	⊕⊕⊝⊝ **low**	
**Quality of Life** **(Social Score)**	The mean score ranged across control groups from −8.13 to 8.63.	The mean score in the intervention groups was 12.76 (−0.09 to 23.10).	/	323	⊕⊕⊝⊝ **low**	
**Quality of Life** **(Environment Score)**	The mean score ranged across control groups from −5.00 to 0.25.	The mean score in the intervention groups was 4.82 (0.18 to 10.57).	/	323	⊕⊕⊝⊝ **low**	
**Social Function**	The mean score ranged across control groups from −1.48 to 3.54.	The mean score in the intervention groups was 4.85 (2.74 to 7.57).	/	289	⊕⊕⊕⊝ **moderate**	
**Cognition**	The mean score ranged across control groups from −0.07 to 1.40.	The mean score in the intervention groups was 1.39 (1.07 to 1.77).	/	132	⊕⊕⊝⊝ **low**	
**Depression**	The mean score ranged across control groups from −1.63 to 2.33.	The mean score in the intervention groups was 2.71 (0.70 to 4.83).	/	269	⊕⊕⊕⊝ **moderate**	
**Anergia**	The mean score ranged across control groups from v2.00 to 1.03.	The mean score in the intervention groups was 2.34 (1.13 to 3.20).	/	109	⊕⊕⊝⊝ **low**	
**Side Effects**	The mean score ranged across control groups from −0.20 to 0.10.	The mean score in the intervention groups was 0.60 (0.30 to 0.90).	/	149	⊕⊕⊝⊝ **low**	
**Extrapyramidal Symptoms**	The mean score ranged across control groups from 0.60 to 0.80.	The mean score in the intervention groups was 0.20 (−0.10 to 0.50).	/	106	⊕⊕⊝⊝ **low**	

*The basis for the assumed risk (e.g. the median control group risk across studies) is provided in footnotes. The corresponding risk (and its 95% confidence interval) is based on the assumed risk in the comparison group and the relative effect of the intervention (and its 95% CI).

CI, Confidence interval; RR, Risk Ratio; GRADE, Grading of Recommendations Assessment, Development and Evaluation.

GRADE Working Group grades of evidence.

High quality: Further research is very unlikely to change our confidence in the estimate of effect.

Moderate quality: Further research is likely to have an important impact on our confidence in the estimate of effect and may change the estimate.

Low quality: Further research is very likely to have an important impact on our confidence in the estimate of effect and is likely to change the estimate.

Very low quality: We are very uncertain about the estimate.

### Outcomes

#### Mind–Body Exercises *vs*. Treatment as Usual

**Figure 3:**
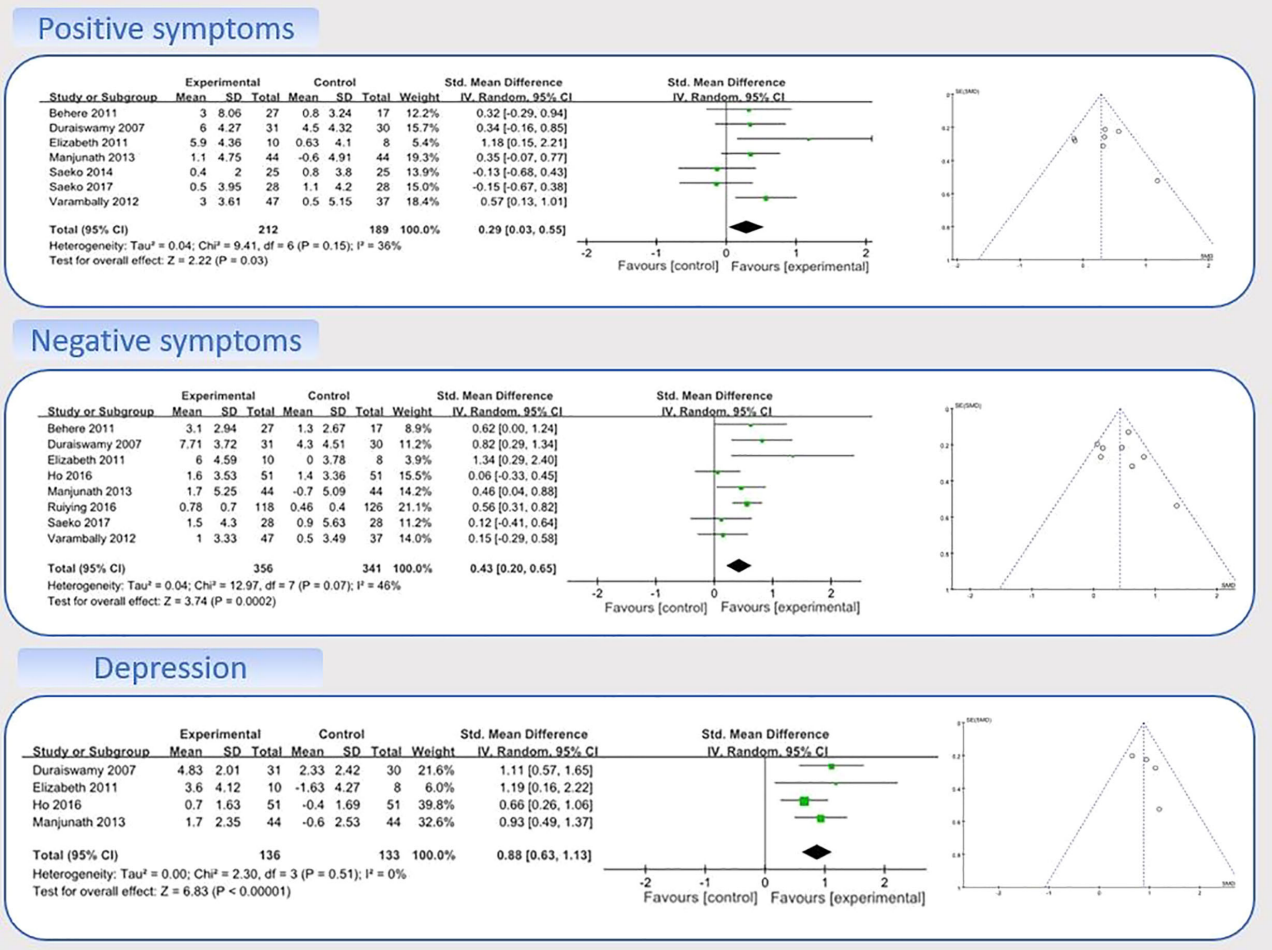
Forest Plot and Funnel Plot for Positive Symptoms, Negative Symptoms, and Depression.

Nine studies with data on the effects of MBEs on positive symptoms were entered into the model 1 (22–24, 36, 39–42, 45). A sensitivity analysis was performed to determine consistency of the effects of MBEs on positive symptoms. By checking both the visually asymmetrical Funnel Plot (**Figure 3**) and the Egger’s Regression Test (Egger’s regression intercept = 2.70, *p* = 0.17), two studies were removed (SMD = 0.34) (24, 42). After their removal, the funnel plot of the remaining studies showed a symmetrical Funnel plot (Egger’s regression intercept = 1.43, *p* = 0.56).

For the meta-analysis in the remaining seven studies, compared with the control group, the aggregated results showed a significant benefit in favor of MBEs on positive symptoms (SMD = 0.29; 95% CI 0.03 to 0.55; I² = 36%; *p* = 0.03; **Figure 3**).

Nine studies with data on the effects of MBEs on negative symptoms were entered into the model 1 (22–24, 36, 39–42, 45). A sensitivity analysis was performed to determine consistency of the effects of MBEs on negative symptoms. By checking both the visually asymmetrical Funnel Plot (**Figure 3**) and the Egger’s Regression Test (Egger’s regression intercept = 0.26, *p* = 0.88), one study was then removed (SMD = −0.19) (41). After that, the remaining eight studies showed a symmetrical Funnel plot (Egger’s regression intercept = 0.88, *p* = 0.58). Meta-analysis showed that compared with the control group, the aggregated results showed a benefit in favor of MBEs on negative symptoms (SMD = 0.43; 95% CI 0.20 to 0.65; I² = 46%; *p* = 0.0002; **Figure 3**).

Four studies with data on the effects of MBEs on depression were entered into the model 2 (22, 24, 36, 40). Compared with the control group, the aggregated results showed a significant benefit in favor of MBEs on depression (SMD = 0.88; 95% CI 0.63 to 1.13; I² = 0%; *P* <0.00001; **Figure 3**).

Compared with usual treatment, there were moderately significant effects in favor of MBE intervention on improving anergia (*p*<0.0001) and side effects (*p* = 0.007). However, no significant effects were found on general psychopathology (*p* = 0.18), social function (*p* = 0.18), cognition (*p* = 0.20), quality of life (physical score: *p* = 0.16), (psychological score: *p* = 0.16), (social score: *p* = 0.23), (environment score: *p* = 0.37), and extrapyramidal symptoms (*p* = 0.13).

#### Meta-Regression

**Figure 4:**
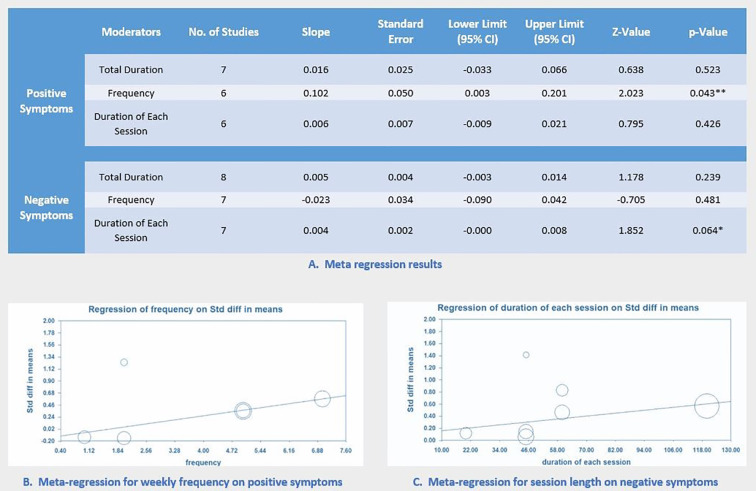
Meta-Regression of Intervention Factors for Improving Symptoms.

For both positive and negative symptoms, multiple separate meta-regressions were performed for total minutes, weekly frequency, and MBE session length. Results showed that weekly frequency was significantly correlated with improved positive symptoms (*p* = 0.04; **Figure 4**). Notably, session length was marginally correlated with improved negative symptoms (*p* = 0.06; **Figure 4**). All results of meta-regression are presented in **Figure 4**.

## Discussion

This meta-analysis systematically evaluated emerging evidence regarding the effects of MBEs on multiple health outcomes for individuals with schizophrenia. Results of the current review indicate that MBEs (primarily including Tai Chi and Yoga) may have beneficial effects for improving disease-specific outcomes (positive symptom, negative symptom and depression). Such promising results suggest that MBEs could be an effective complementary therapy for symptomatic management of schizophrenia. More specifically, weekly frequency has shown to be positively associated with improved positive symptoms, while session length is marginally associated with negative symptoms with non-significant level. Notably, findings of this meta-analytic paper appear to be consistent with two previous reviews on negative symptoms (26, 28), but not positive symptoms.

In the early literature, MBEs are defined as mild to moderate-intensity of exercise modality where practitioners need to perform physical movement at slow pace while integrated with mental focus and relaxation, meditative state of mind, and deep abdominal breathing (46). Such nature of MBEs has been extensively investigated, suggesting that these unique exercise modalities have beneficial effects for mood regulation in healthy populations and treating disease-specific outcomes among individuals with psychiatric disorders, especially negative emotion (*e.g.*, anxiety and depression). Therefore, it seems to be reasonable to observe improved negative symptoms of schizophrenia in this systematic review.

Prefrontal and temporal cortex abnormalities have been shown to be connected with symptoms (negative emotion, inattention, dysfunction in cognition) of schizophrenic patients (47–49). Biological mechanism remains largely unknown about how MBEs affect symptoms of schizophrenia. Some researchers proposed that these positive results may be attributed to Tai Chi-induced change in brain intrinsic cortical structure and function (50, 51). Early imaging studies by Wei et al. (50) indicated that Tai Chi training was associated with increased cortical thickness in brain regions related to executive functions (50), as well as decreased functional homogeneity in dorsolateral prefrontal cortex that potentially optimizes locally functional organization (51). Such Tai Chi-induced change in the prefrontal lobes of the elderly may be a possible explanation for the observed positive effects for symptoms of schizophrenia in the current review. Additionally, as mentioned previously, meditative stage of mind as an essential element of MBEs needs to be achieved while performing physical movement in coordination with breathing control and mental focus. Meditation alone as an intervention program has been extensively investigated, suggesting that it could positively induce cortical change in the ACC, prefrontal cortex, posterior cingulate cortex, and insula (52–57). These areas were regarded as core regions for self-regulation of attention (52, 53), emotion (54, 56), and awareness (55, 57). Thus, it is plausible that the meditative component of MBEs might play an important role to alleviate symptoms of schizophrenia by improving brain structure and function.

Glucose metabolic abnormality was highly prevalent in patients with schizophrenia. This abnormality is mainly processed by a decrease in cerebral insulin receptors’ (*β*-subunit) expression (58), signal transduction protein Akt1 activity (59) and insulin-degrading enzyme neuronal expression (60). Different patterns of regional glucose metabolism are related to different schizophrenia syndromes: psychomotor poverty with left prefrontal and superior parietal metabolic activity, reality distortion with left temporal lobule, and disorganization with left inferior parietal lobule (61). Recently, Huocheng et al. reported that aerobic exercise has beneficial effects for improving glucose metabolism in the medial frontal gyrus (MFG), which involves executive and visuospatial attentional functions (62). A recent study found that one-year aerobic exercise improved not only the glucose metabolism but also psychiatric symptoms (63). Thus, MBEs as typical type of aerobic exercise modalities may have the potential to improve symptoms of schizophrenia through regulating glucose metabolism.

Several limitations should be acknowledged while interpreting our findings. Firstly, several included studies had a small sample size with incomplete information, which limited our capability to conduct subgroup analyses and more comprehensive data exploration of moderators. Secondly, MBEs were offered as adjunctive treatments of existing interventions but not mono-therapy in most studies. It is difficult to determine whether the positive result is due to MBEs only, the synergistic intervention, or conventional treatment received.

### Implication

This study showed that MBEs are beneficial for schizophrenia as an adjunctive treatment. These benefits can be seen in various aspects of schizophrenia prognosis and exist throughout a person’s life. In addition, MBE can also reduce the potential risks of antipsychotics (*i.e.*, hesitation, retention, and transient leukopenia) (10, 11), which are critical to the patients’ quality of life and well-being. Therefore, a comprehensive intervention of pharmacological and non-pharmacological treatment (*i.e.*, MBE) should be considered for patients with schizophrenia. In addition, it is necessary for the therapists who teach MBEs to receive training in mental health disorders to sensitize them to the needs of patients. Psychiatrist should work closely with therapists so that they can meet the needs of patients at any time.

## Conclusions

This meta-analytic review of the existing literature suggests that MBEs are effective interventions to improve symptoms of schizophrenia. These findings provide safe and accessible therapy to existing mainstream treatment (antipsychotic drugs and psychotherapy), and clinicians should consider using MBEs as complementary treatment for schizophrenia.

In the future, more strictly-designed RCTs with larger scale are warranted to examine the therapeutic effects and potential mechanism of MBEs for schizophrenia. Additionally, it is also needed to explore how other types of MBEs (*i.e.*, Tai Chi Chuan, Qigong) influence the symptoms of schizophrenia so as to advance the understanding of general benefits of the varied forms of MBEs. Since impaired cognition is one of the main symptoms for schizophrenia, researchers should investigate the effect of MBEs on cognitive improvement of schizophrenia.

## Data Availability Statement

The original contributions presented in the study are included in the article/supplementary material; further inquiries can be directed to the corresponding author.

## Author Contributions

G-XW and XZ designed the study. QY wrote the protocol. QY, LY, and KI managed the literature searches and analyses. QY, PL, and LS undertook the statistical analysis, and G-XW and QY wrote the first draft of the manuscript. All authors contributed to the article and approved the submitted version.

## Funding

This work was supported by the Key Laboratory of Mental Health and Key Laboratory of Behavioral Science, Institute of Psychology, Chinese Academy of Sciences by the Scientific Foundation of Institute of Psychology, Chinese Academy of Sciences (Grant No. Y9CX402008) and National Natural Science Foundation of China (Grant No. 31671163; Grant No. 31871115).

## Conflict of Interest

The authors declare that the research was conducted in the absence of any commercial or financial relationships that could be construed as a potential conflict of interest.

## References

[1] Moreno-Küstner B Martín C Pastor L Prevalence of psychotic disorders and its association with methodological issues. A systematic review and meta-analyses. PloS One (2018) 13(4):e0195687. 10.1371/journal.pone.0195687 29649252 PMC5896987

[2] van Os J KS Schizophrenia. Lancet (2009); (9690) 374:635–45. 10.1016/s0140-6736(09)60995-8 19700006

[3] Dominguez Mde G Viechtbauer W Simons CJ van Os J Krabbendam L Are psychotic psychopathology and neurocognition orthogonal? A systematic review of their associations. Psychol Bull (2009) 135(1):157–71. 10.1037/a0014415 19210058

[4] Hadjulis M Margariti M Lazaridou M Angelidis GF Fotopoulos V Markaki L Clinical guidelines for the management of schizophrenia: Pharmacological and psychological intervent ions (III). Psychiatriki (2018) 29(4):303–15. 10.22365/jpsych.2018.294.303 30814040

[5] Wu EQ Shi L Birnbaum H Hudson T Kessler R Annual prevalence of diagnosed schizophrenia in the USA: a claims data analysis approach. Psychol Med (2006) 36(11):1535–40. 10.1017/s0033291706008191 16907994

[6] Stahl SM Buckley PF Negative symptoms of schizophrenia: a problem that will not go away. Acta Psychiat Scand (2007) 115(1):4–11. 10.1111/j.1600-0447.2006.00947.x 17201860

[7] Pitkanen A Hatonen H Kuosmanen L Valimaki M Individual quality of life of people with severe mental disorders. J Psychiatr Ment Health Nurs (2009) 16(1):3–9. 10.1111/j.1365-2850.2008.01308.x 19192080

[8] Zeidler J Slawik L Fleischmann J Greiner W The costs of schizophrenia and predictors of hospitalisation from the statutory health insurance perspective. Health Econ Rev (2012) 2(1):9. 10.1186/2191-1991-2-9 22828440 PMC3464783

[9] Sheitman BB Lieberman JA Kaplan RD Szechtman H Franco S Szechtman B The natural history and pathophysiology of treatment resistant schizophrenia. Schizophr Res (1998) 32(3-4):143–50. 10.1016/0920-9964(93)90037-j 9793867

[10] Gaebel W Stricker J Riesbeck M The long-term antipsychotic treatment of schizophrenia: A selective review of clinical guidelines and clinical case examples. Schizophr Res (2019) S0920–9964(19)30484–0. 10.5414/CP203704 31806527

[11] Yasui-Furukori N Shimoda K Recent trends in antipsychotic polypharmacy in the treatment of schizophrenia. (2020). 10.1002/npr2.12127 PMC7722682 32672006

[12] Paton C Esop R Young C Taylor D Obesity, dyslipidaemias and smoking in an inpatient population treated with antipsychotic drugs. Acta Psychiat Scand (2004) 110(4):299–305. 10.1111/j.1600-0447.2004.00372.x 15352932

[13] Vancampfort DKJ De Hert M Wv R Seppe D Katrien M Joseph P Cardiometabolic effects of physical activity interventions for people with schizophrenia. Acta Psychiat Scand (2008) 14(3):388–98. 10.1111/j.1600-0447.2007.01032.x

[14] Gorczynski P FG Exercise therapy for schizophrenia. Cochrane Database Syst Rev (2010) 5:Cd004412. 10.1002/14651858.CD004412.pub2 PMC4164954 20464730

[15] Vancampfort D Knapen J Probst M Van Winkel R Peuskens J Maurissen K The therapeutic value of physical exercise for people with schizophrenia. Lancet (London England) (2010) 52(8):565–74. 10.1016/s0140-6736(09)60995-8 20697996

[16] Holley J Crone D Tyson P Lovell G The effects of physical activity on psychological well-being for those with schizophrenia: A systematic review. Br J Clin Psychol (2011) 50(1):84–105. 10.1348/014466510x496220 21332522

[17] Vancampfort D Knapen J Probst M Scheewe T Remans S De Hert M A systematic review of correlates of physical activity in patients with schizophrenia. Acta Psychiat Scand (2012) 125(5):352–62. 10.1111/j.1600-0447.2011.01814.x 22176559

[18] Zou L YA Li C Wei GX Chen KW Kinser PA Effects of meditative movements on major depressive disorder: a systematic review and meta-analysis of randomized controlled trials. J Clin Med (2018) 7(8):195. 10.3390/jcm7080195 PMC6111244 30071662

[19] Wu WW Kwong E Lan XY Jiang XY The Effect of a Meditative Movement Intervention on Quality of Sleep in the Elderly: A Systematic Review and Meta-Analysis. J Altern Complement Med (New York NY) (2015) 21(9):509–19. 10.1089/acm.2014.0251 26120865

[20] Bower JE Irwin MR Mind-body therapies and control of inflammatory biology: A descriptive review. Brain Behav Immun (2016) 51:1–11. 10.1016/j.bbi.2015.06.012 26116436 PMC4679419

[21] Chen S Zhang Y Wang YT Liu XL Traditional Chinese Mind and Body Exercises for Promoting Balance Ability of Old Adults: A Systematic Review and Meta-Analysis. Evidence-Based Complement Altern Med eCAM (2016) 2016:7137362. 10.1155/2016/7137362 PMC5136631 27990168

[22] Duraiswamy G TJ Nagendra HR Gangadhar BN Yoga therapy as an add-on treatment in the management of patients with schizophrenia–a randomized controlled trial. Acta Psychiat Scand (2007) 116(3):226–32. 10.1111/j.1600-0447.2007.01032.x 17655565

[23] Behere RV Arasappa R Jagannathan A Varambally S Venkatasubramanian G Thirthalli J Effect of yoga therapy on facial emotion recognition deficits, symptoms and functioning in patients with schizophrenia. Acta Psychiat Scand (2011) 123(2):147–53. 10.1111/j.1600-0447.2010.01605.x 20846271

[24] Ho RT Fong TC Wan AH Au-Yeung FS Wong CP Ng WY A randomized controlled trial on the psychophysiological effects of physical exercise and Tai-chi in patients with chronic schizophrenia. Schizophr Res (2016) 171(1-3):42–9. 10.1016/j.schres.2016.01.038 26822592

[25] Zheng W Li Q Lin J Xiang Y Guo T Chen Q Tai Chi for Schizophrenia: A Systematic Review. Shanghai Arch Psychiatry (2016) 28(4):185–94. 10.11919/j.issn.1002-0829.216051 PMC5434269 28638191

[26] Sabe M Sentissi O Kaiser S Meditation-based mind-body therapies for negative symptoms of schizophrenia: Systematic review of randomized controlled trials and meta-analysis. Schizophr Res (2019) 212:15–25. 10.1016/j.schres.2019.07.030 31378557

[27] Vogel JS van der Gaag M Slofstra C Knegtering H Bruins J Castelein S The effect of mind-body and aerobic exercise on negative symptoms in schizophrenia: A meta-analysis. Psychiatry Res (2019) 279:295–305. 10.1016/j.psychres.2019.03.012 30879703

[28] Li J Shen J Wu G Tan Y Sun Y Keller E Mindful exercise versus non-mindful exercise for schizophrenia: A systematic review and meta-analysis of randomized controlled trials. Complement Ther Clin Pract (2018) 32:17–24. 10.1016/j.ctcp.2018.04.003 30057047

[29] Moher DLA Teztlaff J Szeszko PR Hodgkinson CA Robinson DG Derosse P The PRISMA Group: Preferred Reporting Items for Systematic Reviews and Meta-Analyses: The PRISMA Statement. Biol Psychol (2009) 51(1):1–7. 10.1016/j.biopsycho.2007.10.011

[30] Higgins GJ Gorczynski P Faulkner G Cochrane Handbook for Systematic Reviews of Interventions.Version 5.1.0. Cochrane Database Syst Rev (2011) 5:Cd004412. 10.1002/14651858.CD004412.pub2

[31] Goldet G Howick J Understanding GRADE: an introduction. J Evid Based Med (2013) 6(1):50–4. 10.1111/jebm.12018 23557528

[32] Cohen J Statistical Power Analysis for the Behavioral Sciences. USA: Academic Press (1988). 10.1016/C2013-0-10517-X

[33] van Tulder MFA Bombardier C Bouter LE Editorial Board of the Cochrane Collaboration Back Review Group: Updated method guidelines for systematic reviews in the cochrane collaboration back review group. Spine (2003) 28:1290–9. 10.1097/01.BRS.0000065484.95996.AF 12811274

[34] Higgins JP Thompson SG Deeks JJ Altman DG Measuring inconsistency in meta-analyses. BMJ (Clin Res ed) (2003) 327(7414):557–60. 10.1136/bmj.327.7414.557 PMC192859 12958120

[35] Sarah KE Lisa JP Wood SJ Uren J Mallawaarachchi SR Cotton SM Depressive psychopathology in first-episode schizophrenia spectrum disorders: a systematic review, meta-analysis and meta-regression. J Clin Med (2019) 2019(8):1–12. 10.3390/jcm7080195 31524121

[36] Elizabeth V Stephen L Yoga therapy as an adjunctive treatment for schizophrenia: a randomized, controlled pilot study. J Altern Complement Med (New York NY) (2011) 17(7):601–7. 10.1089/acm.2010.0075 21711202

[37] Bhatia T Agarwal A Shah G Wood J Richard J Gur RE Adjunctive cognitive remediation for schizophrenia using yoga: an open, non-randomized trial. Acta Neuropsychiatr (2012) 24(2):91–100. 10.1111/j.1601-5215.2011.00587.x 22661830 PMC3361970

[38] Rainbow TH Ho FS Yeung A Tai-Chi for Residential Patients with Schizophrenia on Movement coordination, negative symptoms, and functioning: a pilot randomized controlled trial. Evidence-Based Complement Altern Med (2012) 2012:923925. 10.1155/2012/923925 PMC3524789 23304224

[39] Varambally SGB Thirthalli J Brewer JA Worhunsky PD Gray JR Tang YY Therapeutic eﬃcacy of add-on yogasana intervention in stabilized outpatient schizophrenia: randomized controlled comparison with exercise and waitlist. Proc Natl Acad Sci United States America (2012) 2012(54):227–32. 10.1073/pnas.1112029108 PMC3512358 23226845

[40] Manjunath RB Varambally S Thirthalli J Basavaraddi IV Gangadhar BN Efficacy of yoga as an add-on treatment for in-patients with functional psychotic disorder. Indian J Psychiatry (2013) 55(Suppl 3):S374–378. 10.4103/0019-5545.116314 PMC3768215 24049202

[41] Saeko I Takefumi S Hiroyuki U Juri S Keiichi T Yasuo F Effects of weekly one-hour Hatha yoga therapy on resilience and stress levels in patients with schizophrenia-spectrum disorders: an eight-week randomized controlled trial. J Altern Complement Med (New York NY) (2014) 20(11):823–30. 10.1089/acm.2014.0205 25364946

[42] Kang R Wu Y Li Z Jiang J Gao Q Yu Y Effect of Community-Based Social Skills Training and Tai-Chi Exercise on Outcomes in Patients with Chronic Schizophrenia: A Randomized, One-Year Study. Psychopathology (2016) 49(5):345–55. 10.1159/000448195 27584836

[43] Funda K Mine E The Effect of Yoga on Functional Recovery Level in Schizophrenic Patients. Arch Psychiatr Nurs (2016) 30(6):761–7. 10.1016/j.apnu.2016.07.010 27888972

[44] Bhatia T Mazumdar S Wood J He F Gur RE Gur RC A randomised controlled trial of adjunctive yoga and adjunctive physical exercise training for cognitive dysfunction in schizophrenia. Acta Neuropsychiatr (2017) 29(2):102–14. 10.1017/neu.2016.42 PMC5303681 27514629

[45] Saeko I Hiroyuki U Yuya M Hideaki T Maki N Kenichi T Effects of chair yoga therapy on physical fitness in patients with psychiatric disorders: A 12-week single-blind randomized controlled trial. J Psychiatr Res (2017) 94:194–201. 10.1016/j.jpsychires.2017.07.015 28750232

[46] Stanton R Happell B A systematic review of the aerobic exercise program variables for people with schizophrenia. Curr sports Med Rep (2014) 13(4):260–6. 10.1249/jsr.0000000000000069 25014392

[47] Cynthia JG Martha ES Prefrontal cortex, negative symptoms, and schizophrenia: an MRI study. Psychiatry Res: Neuroimaging Section (2001) 108:65–78. 10.1016/S0925-4927(01)00109-3 PMC2845854 11738541

[48] Szeszko PR HC Robinson DG Derosse P Bilder RM Lencz T DISC1 is associated with prefrontal cortical gray matter and positive symptoms in schizophrenia. Biol Psychol (2008) 79(1):103–10. 10.1016/j.biopsycho.2007.10.011 PMC2623247 18078707

[49] Murray AJ Woloszynowska-Fraser MU Ansel-Bollepalli L Cole KL Foggetti A Crouch B Parvalbumin-positive interneurons of the prefrontal cortex support working memory and cognitive flexibility. Sci Rep (2015) 5:16778. 10.1038/srep16778 26608841 PMC4660359

[50] Wei GX Xu T Fan FM Dong HM Jiang LL Li HJ Can Taichi reshape the brain? A brain morphometry study. PloS One (2013) 8(4):e61038. 10.1371/journal.pone.0061038 23585869 PMC3621760

[51] Wei GX Dong HM Yang Z Luo J Zuo XN Tai Chi Chuan optimizes the functional organization of the intrinsic human brain architecture in older adults. Front Aging Neurosci (2014) 6:74:74. 10.3389/fnagi.2014.00074 24860494 PMC4029006

[52] Holzel BK Ott U Hempel H Hackl A Wolf K Stark R Differential engagement of anterior cingulate and adjacent medial frontal cortex in adept meditators and non-meditators. Neurosci Lett (2007) 421(1):16–21. 10.1016/j.neulet.2007.04.074 17548160

[53] Tang YY Ma Y Fan Y Feng H Wang J Feng S Central and autonomic nervous system interaction is altered by short-term meditation. Proc Natl Acad Sci United States America (2009) 106(22):8865–70. 10.1073/pnas.0904031106 PMC2690030 19451642

[54] Goldin PR Gross JJ Effects of mindfulness-based stress reduction (MBSR) on emotion regulation in social anxiety disorder. Emotion (Washington DC) (2010) 10(1):83–91. 10.1037/a0018441 PMC4203918 20141305

[55] Brewer JA WP Gray JR Tang YY Weber J Kober H Meditation experience is associated with differences in default mode network activity and connectivity. Proc Natl Acad Sci United States America (2011) 108(50):20254–9. 10.1073/pnas.1112029108 PMC3250176 22114193

[56] Desbordes G Negi LT Pace TW Wallace BA Raison CL Schwartz EL Effects of mindful-attention and compassion meditation training on amygdala response to emotional stimuli in an ordinary, non-meditative state. Front Hum Neurosci (2012) 6:292. 10.3389/fnhum.2012.00292 23125828 PMC3485650

[57] Hasenkamp W Barsalou LW Effects of meditation experience on functional connectivity of distributed brain networks. Front Hum Neurosci (2012) 6:38. 10.3389/fnhum.2012.00038 22403536 PMC3290768

[58] Emamian ES Hall D Birnbaum MJ Karayiorgou M Gogos JA Convergent evidence for impaired AKT1-GSK3beta signaling in schizophrenia. Nat Genet (2004) 36(2):131–7. 10.1038/ng1296 14745448

[59] Zhao Z Ksiezak-Reding H Riggio S Haroutunian V Pasinetti GM Insulin receptor deficits in schizophrenia and in cellular and animal models of insulin receptor dysfunction. Schizophr Res (2006) 84(1):1–14. 10.1016/j.schres.2006.02.009 16581231

[60] Ronald K HS SF Three clinical syndromes of schizophrenia in untreated subjects: relation to brain glucose activity measured by position emission tomography (PET). Schizophr Res (1993) 11(1):47–54. 10.1016/0920-9964(93)90037-J 8297804

[61] Liao HC Zhong SG Li P Chen WB Cheng C Wang YG Effects and mechanism of moderate aerobic exercise on impaired fasting glucose improvement. Lipids Health Dis (2015) 14:157. 10.1186/s12944-015-0117-z 26630989 PMC4668668

[62] Porto FH Coutinho AM Pinto AL Gualano B Duran FL Prando S Effects of Aerobic Training on Cognition and Brain Glucose Metabolism in Subjects with Mild Cognitive Impairment. J Alzheimer’s Dis JAD (2015) 46(3):747–60. 10.3233/jad-150033 25835427

[63] Nyboe L Vestergaard CH Moeller MK Lund H Videbech P Metabolic syndrome and aerobic fitness in patients with first-episode schizophrenia, including a 1-year follow-up. Schizophr Res (2015) 168(1-2):381–7. 10.1016/j.schres.2015.07.053 26278336

